# Tungiasis Presenting as Onychomycosis: Probably the First Report of Flea Infestation of the Nail Observed Using Modified Potassium Hydroxide Mount Technique

**DOI:** 10.7759/cureus.2278

**Published:** 2018-03-05

**Authors:** Venkataramana Kandi

**Affiliations:** 1 Department of Microbiology, Prathima Institute of Medical Sciences

**Keywords:** tungiasis, human tungiasis, tunga penetrans, flea, onychomycosis, modified koh mount

## Abstract

Tungiasis is an infection/infestation of the flea *Tunga penetrans*. These are bloodsucking ectoparasitic insects belonging to the phylum Arthropoda, and they do not possess wings, but they have long legs enabling them to jump up to 30 cm high. The fleas are usually present on the skin and in the hair of domestic and wild animals and are prevalent throughout the world. They may also be present in an environment consisting of dry sandy soils, and they infect people who walk barefoot and reside in flea-infested areas. Human tungiasis is both an accidental and zoonotic infection, where the fleas enter the human skin and cause severe morbidity if not properly managed. There are a few reports of human tungiasis, most of which were diagnosed with skin infections. This is a first-of-its-kind observation of fleas in the nail, from a patient who is suspected to be suffering from onychomycosis. The nail in this case was processed differently, by using a modified potassium hydroxide (KOH) mount technique.

## Introduction

Tungiasis is an infestation of the sand flea that belongs to the phylum Arthropoda, class Insecta, and order Siphonaptera. It is commonly referred to as chigoe flea, and *Tunga penetrans* (*T penetrans*) is its scientific name. These fleas are named differently as Jigger, Nigua, Pico/Pio, Bicho de pie (Spain), Bicho de pe (Portugal), Bichodo porco, Pique, Pulga de areia Kuti, Jatecuba, and Sikka in various parts of the world. *T penetrans*, belongs to the genus Tunga and family Tungidae, which contain obligate hematophagous ectoparasites. There are around 14 species of Tunga fleas, and this is also the smallest known flea, measuring up to 1 mm. Along with *T penetrans*, only two other species, *Tunga trimamillata* and *Tunga hexalobulata*, are known to parasitize animals, with *T penetrans* being associated with human infections [1-3]. Tungiasis is highly endemic to Trinidad and Tobago, Nigeria, and Brazil, where 50% people have been noted to be suffering from infections with *T penetrans*. Tungiasis is an ubiquitous infection spread throughout the world and has been reported from more than 88 countries, including South and Central America, Asia, Africa, and Europe. The flea *Tunga penetrans* is native to West Indies, Caribbean islands, other regions of Africa (sub-Saharan Africa, Kenya, Uganda), India, Pakistan, Nepal, and Latin American countries [4]. Tungiasis in humans was first reported in the years following 1500 AD, when sailors with Christopher Columbus suffered skin infections, after they were shipwrecked on an island of Haiti. Fleas responsible for tungiasis have limited jumping/flying abilities (<30 cm), and therefore most human lesions appear on the skin, below the waist, particularly on the legs. Lesions were also reported from other areas of the body, including the hands, elbow, neck, and face [5]. These fleas like a warm and dry environment and are present in the soil under stables, stock farms, beaches, coastal areas, desert soils, and other sandy soils [6]. Pigs, dogs, cats, rats, sheep, cattle, donkeys, monkeys, birds, and elephants are considered as reservoirs of fleas causing tungiasis [7-8]. Reports of human tungiasis have been recently growing, and there is only one report of human tungiasis from India previously [9-10]. This observation, the first of its kind where the insects were observed in the nail, was performed using a modified potassium hydroxide mount (KOH) technique.

## Technical report

Patients usually present to the dermatology department with infections/conditions of the skin, hair, and nails. Most of these lesions are attributed to infection with microorganisms, including bacteria, viruses, fungi, and occasionally parasites. Many other skin lesions are due to allergic/hypersensitive reactions and must be cautiously diagnosed ruling out infectious etiology.

Clinical microbiology laboratories receive samples of skin, hair, and nail, and in most instances the microbiologists mostly look for the presence of fungal elements (yeast cells, filamentous fungi). A potassium hydroxide (KOH) mount is routinely performed to see the presence of fungal elements.

Recently, there have been reports of the occurrence of other ectoparasites in the skin, including the mite, which is the causative agent of scabies. A routine KOH mount may fail to detect the presence of mites, as KOH acts as a keratinolytic and may immobilize the insects and kill them, making them invisible on microscopy. Technical modifications during the preparation and processing of specimens could increase the chances of finding the parasites [11].

This is a report of a 45-year-old female patient, who presented to the dermatology outpatient department (OPD), with complaints of discoloration of nails (disfiguring and blackening), involving both the hands and the toe nails for three months. The dermatologists suspected a case of onychomycosis (tinea unguium) caused by fungus and sent nail clippings both from the toes and the fingers for further microbiological examination and confirmation.

**Figure 1:**
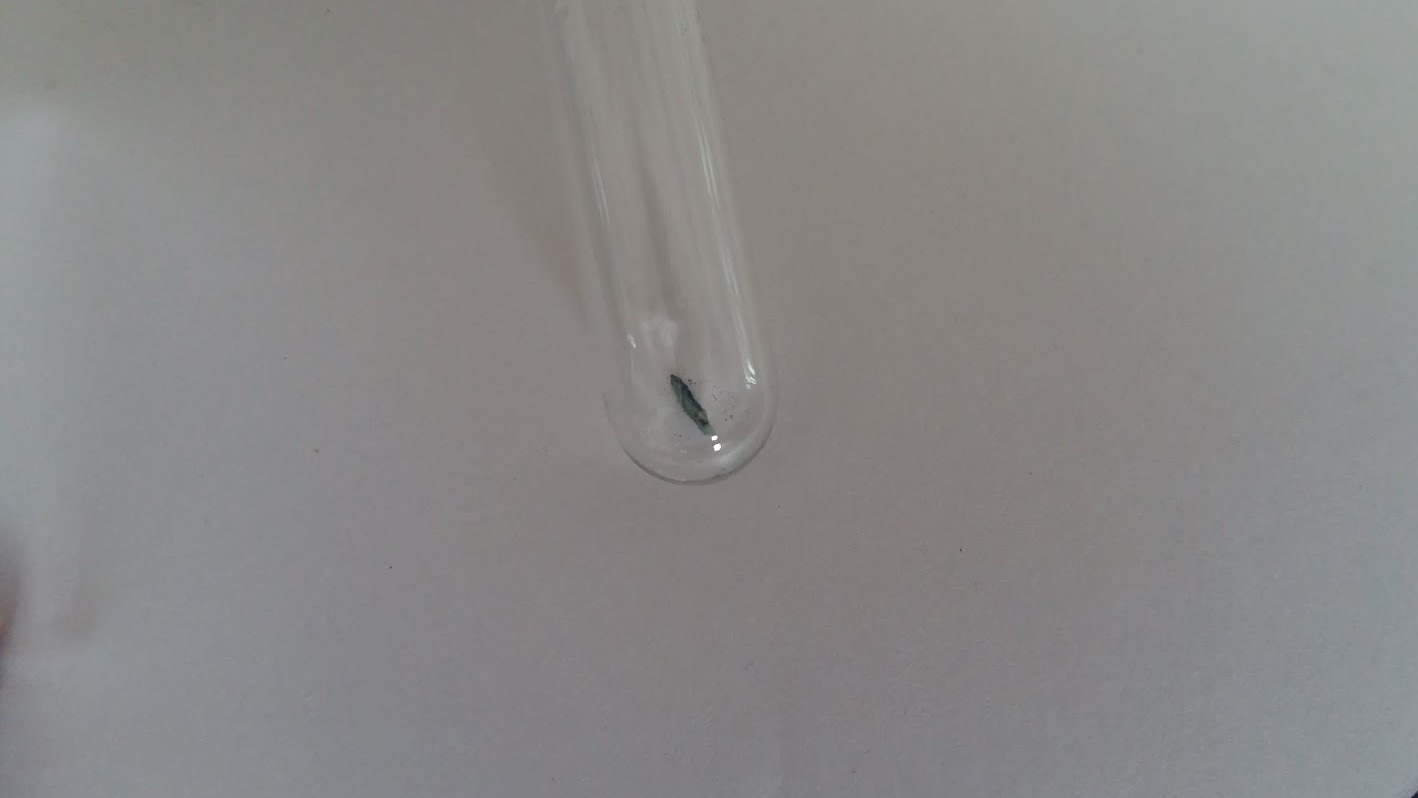
Infected nail clipping seen as black in color

The nail clippings were observed to be black in color and brittle (Figure 1), in contrast to being hard, white to yellowish in color, and crumbling at the edges when there is a fungal infection.

The sample was processed for routine KOH mount (40%) and observed under a low power (10X) and high power (40X) objective of a compound microscope and was found to be negative for fungal elements.

**Figure 2:**
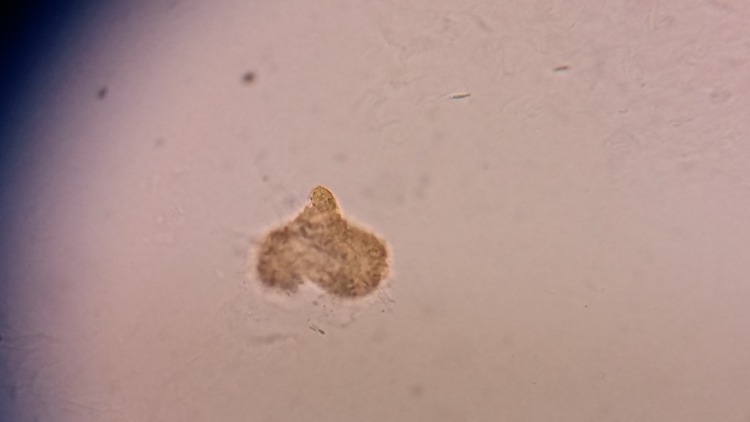
A gravid female flea that is enlarged and releasing eggs

The sample was then processed by using a modified KOH mount technique, which revealed various stages of the life cycle of the flea, probably *T penetrans*. The gravid female flea, which is enlarged and releasing eggs is seen in Figure 2.

**Figure 3:**
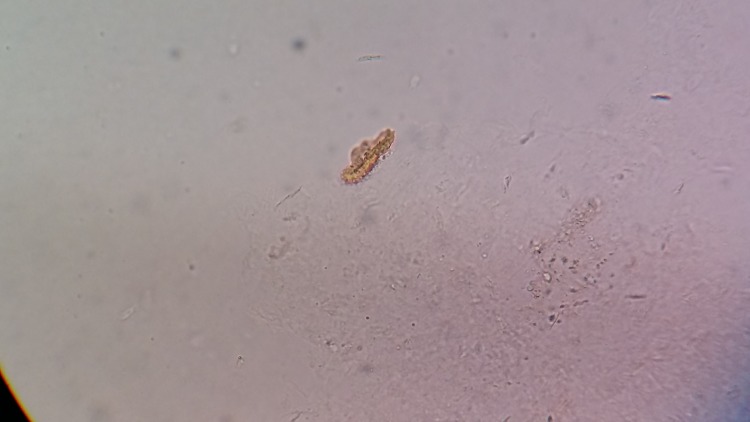
Larval form of a flea after it hatched out of an egg

Figure 3 shows the larval form of the flea after it hatched out of the egg.

**Figure 4:**
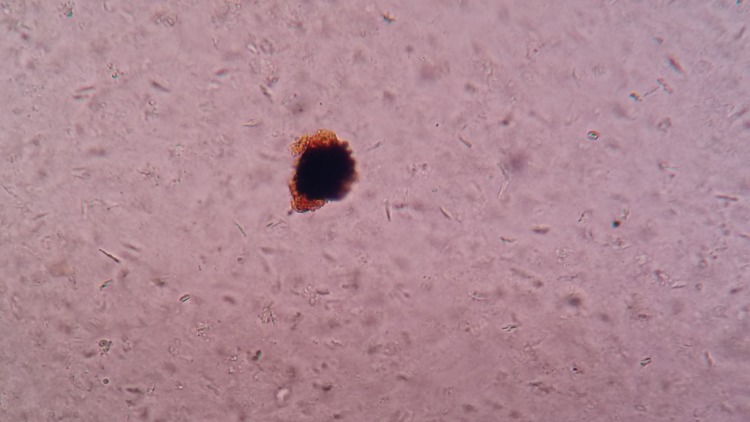
Pupa developing from the larval form

Figure 4 shows the gradual development of the larva into the next stage, the pupa.

**Figure 5:**
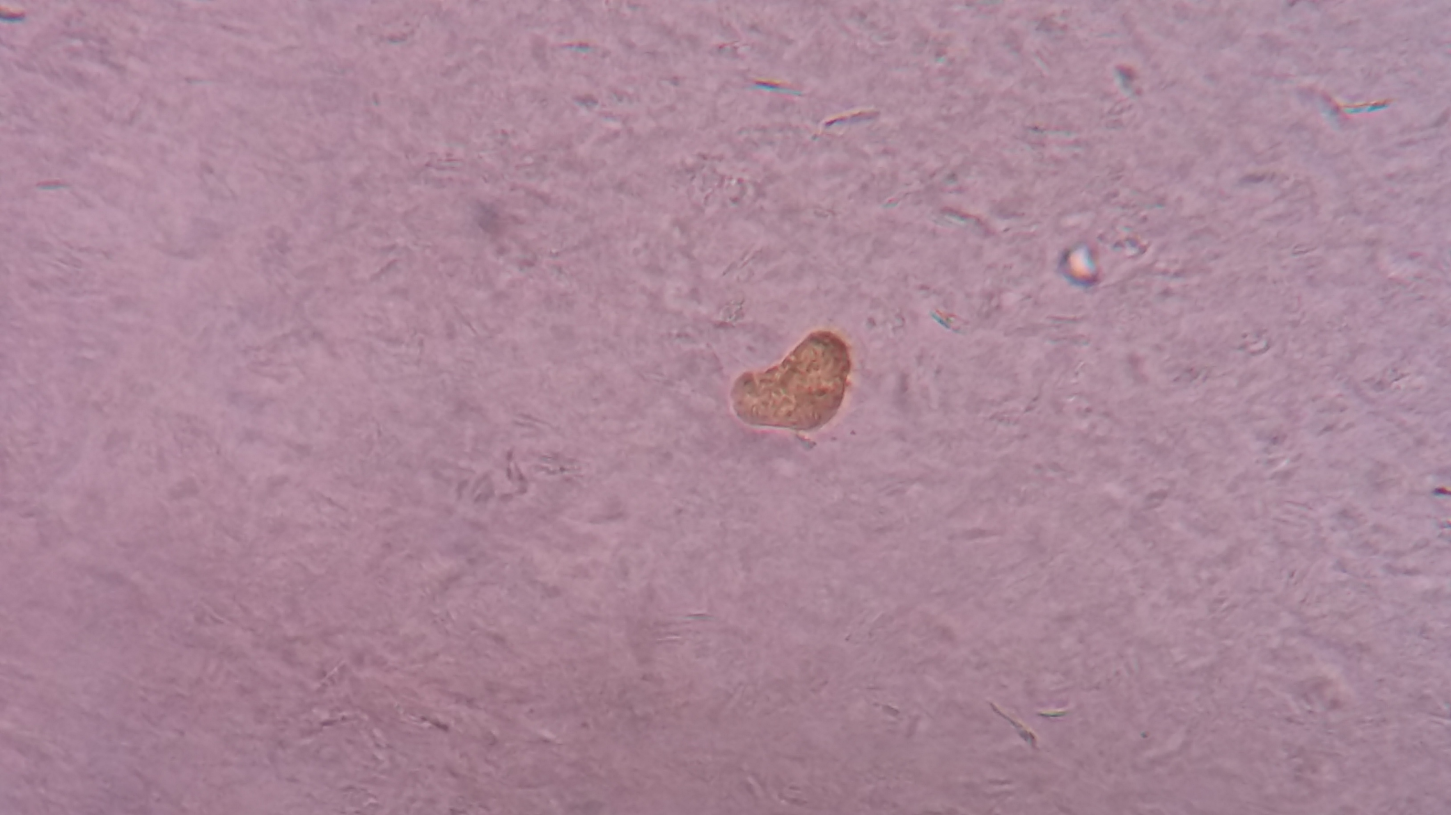
A young adult flea ready to be released from the pupa

Figure 5 shows the pupal form that is ready to release a young adult flea.

**Figure 6:**
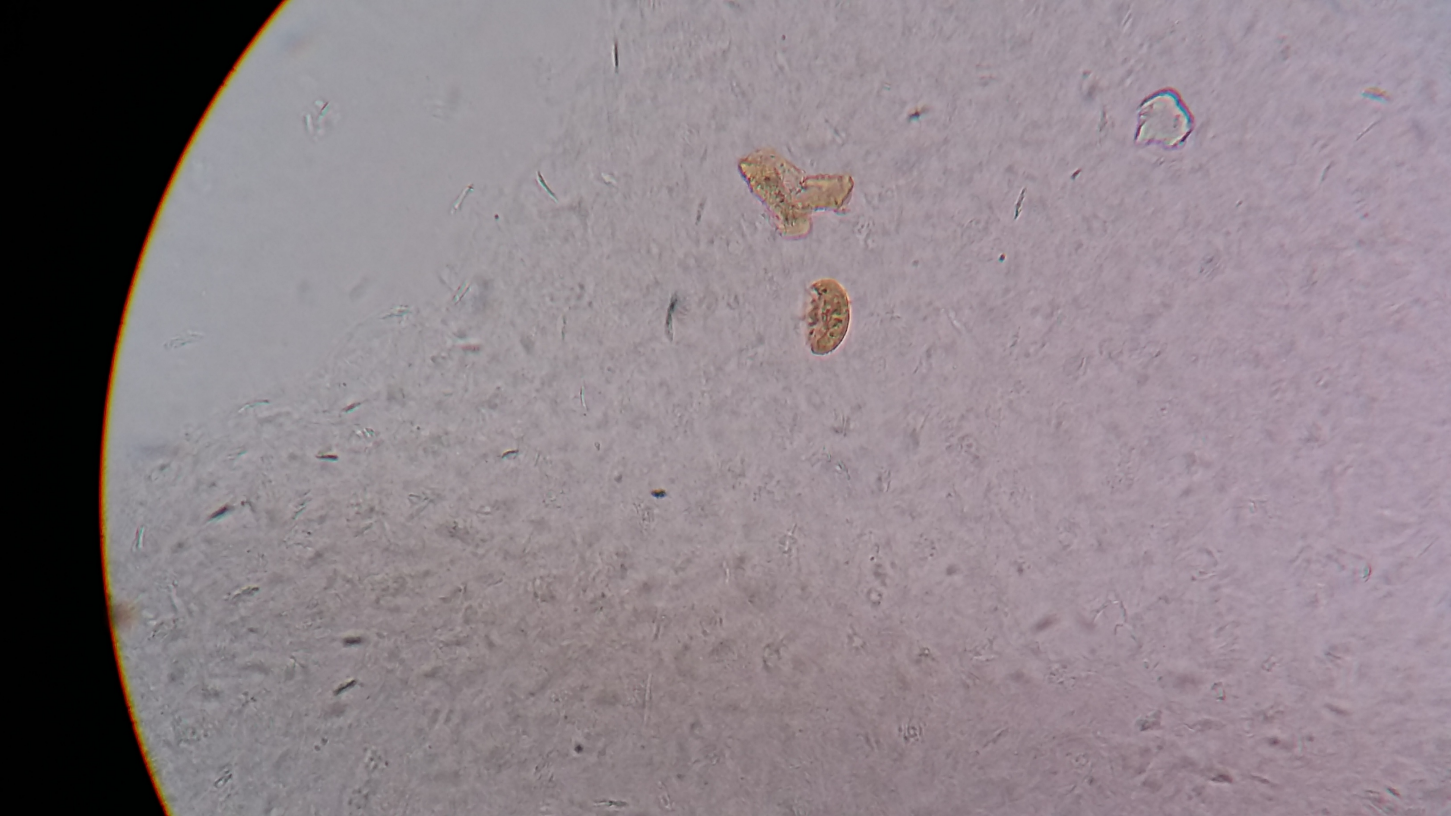
A young adult flea after coming out of the pupa

The young adult flea coming out of the pupa can be seen in Figure 6.

Modified KOH mount technique

KOH is an inorganic, colorless, solid chemical, commonly referred to as caustic potash. It is a strong alkaline substance that shows keratinolytic activity and helps in dissolving the hard tissues. It is used in varied concentrations to dissolve human tissues and observe for microorganisms.

Clinical microbiology laboratories use three different concentrations of KOH: a 10% KOH for observing skin scrapings, a 20% KOH for hair, and a 40% KOH for nails. KOH preparation can be modified to increase the chance of finding microorganisms. Adding di-methyl sulphoxide (solvent) reduces the time of finding microorganisms. To increase the visibility of fungal elements, India ink, Chicago sky blue, and lactophenol cotton blue solution can be added with the KOH preparation.

An infected nail, when it is hard, takes a lot of time to dissolve even in 40% KOH, and if kept for longer periods, the KOH may crystallize making it difficult to observe. KOH, with its corrosive activity, might also cause damage to the microorganisms and other parasitic forms, if any, present in the specimen as noted by a previous research report [12]. To improve the chances of finding both the parasitic forms and the microorganisms, I suggest an alternate and modified KOH preparation technique for nails.

The infected nail clippings should be first placed in normal saline and kept at room temperature for four hours. After this, the nail clippings should be placed in 20% KOH for two hours. A simple mount of the nail material should then be made on a clean and grease-free slide. Then a coverslip should be applied, and the nail should be observed under a low power (10X) and high power (40X) objective of a compound microscope. This procedure reduces the time of dissolving of the nail, does not destroy the microorganisms or parasites present in the specimen, and could be effective in demonstrating the parasites, if any, present in the nail.

## Discussion

Human tungiasis is an ectoparasitic infection, which usually presents as redness of the skin with swelling and very itchy and painful/painless lesions. The fleas burrow themselves under the epidermis and move deeper into the upper dermis, near the blood vessels and feed on blood. The adult female flea lays around 100 eggs (neosome: female flea filled with eggs), which in the course of time hatch into larvae, develop into pupae and later into adults. The burrowed fleas normally die within two weeks, sloughing off the affected skin. The fleas release a substance before sucking the blood, which could be responsible for local allergic reactions. Human skin lesions usually appear as white and swollen affected areas with a black and raised center. The lesions show severe inflammation and ulcerate, and they can result in severe pain and itching. In untreated cases, the lesions can develop secondary bacterial infections, including tetanus, pyogenic infections, and gangrene [12].

Multiple skin lesions resembling the common skin infections caused by other microorganisms can be noted in patients suffering from tungiasis. Careful dermoscopic examinations could increase the chance of diagnosis [13]. Complications of human tungiasis include severe tissue damage, intraepithelial abscess, nail deformation, suppuration, and autoamputation of the affected digits (ainhum) [14].

Tungiasis is more common in people who are poor, walk barefoot, reside in sandy soil conditions, and it is more common during the dry seasons. It is seen in varied age groups with the geriatric age group being more susceptible to infestation [15].

A recent report from India had communicated the occurrence of tungiasis among the troops who were serving in the UN peace keeping forces in Central Africa. This report had also observed that more than 69% of the patients had secondary bacterial infection [16]. Recently, the first case from Nepal was reported, which was also the first one to report infection of the genitals by *T penetrans* [17].

Infection among children could be responsible for severe morbidity as evidenced by a recent research by Wiese S et al., from Kenya, who evaluated the dermatology life quality index (DLQI) of the children suffering from tungiasis. The same study had noted that the severity of infection correlated well with the number of live fleas under the skin and the chronicity of infection [18].

Tungiasis is a potential zoonotic infection transmitted from animals (pigs, dogs, cats, etc.) to humans. The knowledge of infection, associated clinical features, and public health awareness regarding the transmission of tungiasis from household animals among the study population was reported by Mutebi F et al. [19].

Recently, there has been a first-of-its-kind report of tungiasis from a European country, Portugal. This communication sensitizes tungiasis, as a potential infection among the travelers returning from the endemic regions and the importance of physician awareness of its clinical presentation [20].

In this particular case, the clinical presentation was atypical (not involving the skin) with black discoloration of the nail, with the presence of morphological forms resembling the flea, and a routine KOH mount was negative for any fungal elements.

## Conclusions

In the era of emergence and re-emergence of microbial infectious agents, it is important for clinicians and dermatologists to continually update themselves and manage the patients appropriately. Clinical microbiology laboratory personnel need to employ improved and modified laboratory techniques to increase the chances of finding parasites. As noted in the present case, the routine microscopic examination failed to show the presence of parasitic forms, whereas the modified KOH mount technique revealed different stages of the life cycle of the flea.
